# Lymphoepithelioma-like carcinoma of the vulva, an underrecognized entity? Case report with a single inguinal micrometastasis detected by sentinel node technique

**DOI:** 10.1186/1746-1596-6-4

**Published:** 2011-01-10

**Authors:** Hans Bösmüller, Sophie Haitchi-Petnehazy, Christine Gruber, Franz Roithmeier, Wolfgang Stummvoll, Gerald Webersinke

**Affiliations:** 1 Dept. of Pathology, Krankenhaus Barmherzige Schwestern, Linz, Austria; 2 Dept. of Gynecology, Krankenhaus Barmherzige Schwestern, Linz, Austria; 3 Laboratory of Molecular Biology and Tumorcytogenetics, I. Internal dept., Krankenhaus Barmherzige Schwestern, Linz, Austria

## Abstract

This report describes an unusual EBV-negative lymphoepithelioma-like carcinoma of the vulva in a 73-year-old patient. The lesion was localised at the right minor labium and was resected by partial vulvectomy. A synchronous sentinel lymph node biopsy revealed a single micrometastasis in the right inguinal region, which prompted local radiotherapy. Follow-up nine months later showed only slight vulvar atrophy, without signs of local recurrence or distant metastases.

Although lymphoepithelioma-like carcinomas of the skin and the female genital tract are presumed to have a better prognosis than their counterparts in the upper aerodigestive tract, possibly due to earlier detection and therapy, this case documents their potential for early metastasis.

## Background

Lymphoepithelial carcinoma is an aggressive EBV-associated neoplasm of the nasopharyngeal region. Lymphoepithelioma-like carcinoma (LELC) of the skin, however, is a rare condition [1-4] and only three cases have been reported in the vulvar region [5-7]. The same is true for other sites of the female genital tract with some cases described in the uterine cervix [8-14], two reports each in the ovary [15], the endometrium [16,12], and the vagina [17,18], and only one in the Bartholin gland [19]. In contrast to LELC of the nasopharynx, there is usually no evidence for an association with EBV, but cervical LELC is commonly associated with human papilloma virus [20-23]. Although LELC is said to be less aggressive in extranasal sites [1], regional lymph node metastases can occur [24,6]. In this case of a 73-year-old woman, a small inguinal metastasis was detected by sentinel node technique.

## Case Presentation

### Clinical history

The patient had undergone hysterectomy for uterine leiomyomas 20 years ago. At the age of 62, she had been diagnosed with lobular breast carcinoma. She had received adjuvant radiation and endocrine therapy and there were neither local recurrence nor metastases. A gynaecological check-up at the age of 72 years was without pathological findings, but one year later she consulted her gynaecologist because of a weeping painful vulvar lesion. A slightly elevated and eroded area with a diameter of 1.5 cm was detected, localised at the right minor labium with a minimal distance of 2 mm to the clitoris. A small biopsy showed a non-keratinizing poorly differentiated carcinoma NOS. The patient was hospitalised, preoperative computed tomography of the pelvis was inconspicuous and there were no signs of distant metastases. Ultrasonography showed normal sized inguinal lymph nodes, which were not indurated in physical examination. The surface epithelium apart of the lesion was intact, atrophy or lichenoid changes were not seen, so the clinicians decided for partial vulvectomy. Using sentinel technique before surgical intervention a lymph node could be marked in the right inguinal region. Though the frozen section was negative, one micrometastasis of 1 mm was detected in the paraffin embedded material. The postoperative course was normal. Due to the fact that the histological invasion depth of the tumor did not exceed 2 mm, the patient did not receive adjuvant treatment, but underwent radiaton of the right inguinal region with a focal dose of 5000cGy in 25 fractions because of the lymph node micrometastasis. Nine months after treatment she developed pruritus and scaling which disappeared with local steroid therapy, and at the next follow-up visit only minimal local skin atrophy was visible.

### Histopathology and Immunohistochemistry

**Figure 1:**
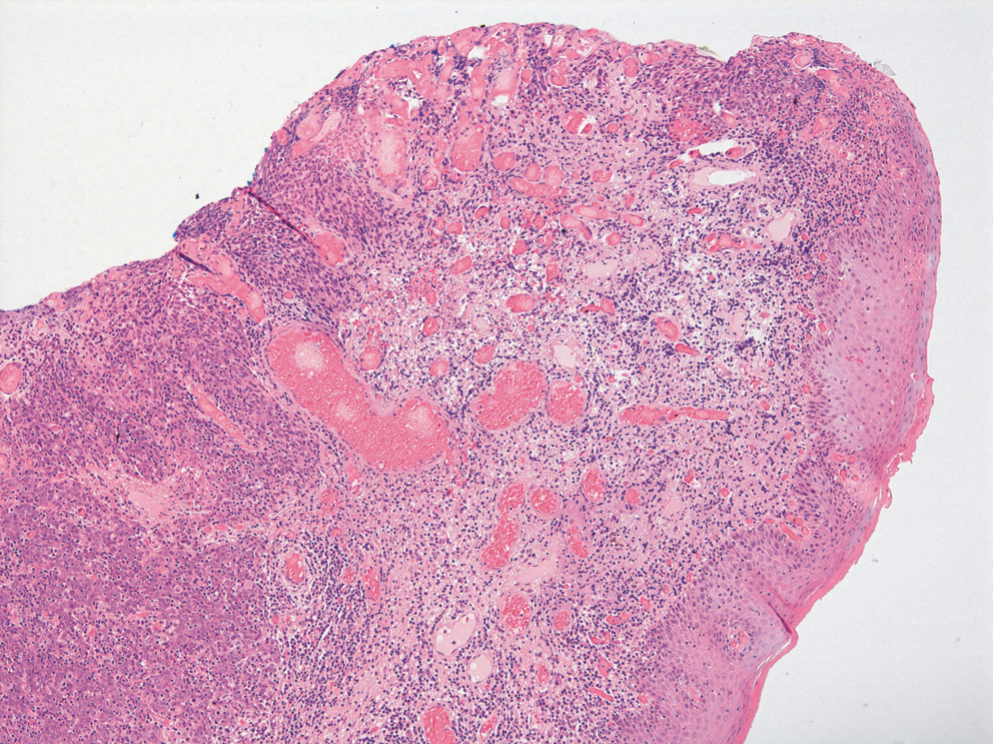
**Right minor labium infiltrated by LELC, H&E 100×**.

**Figure 2:**
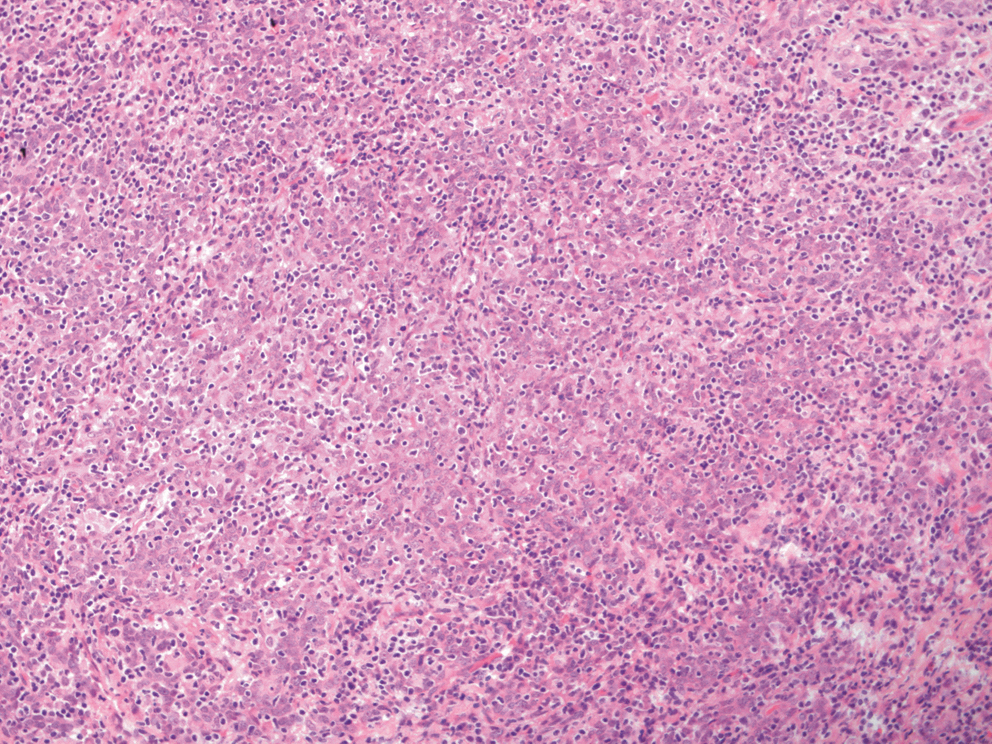
**LELC, H&E 200×**.

**Figure 3:**
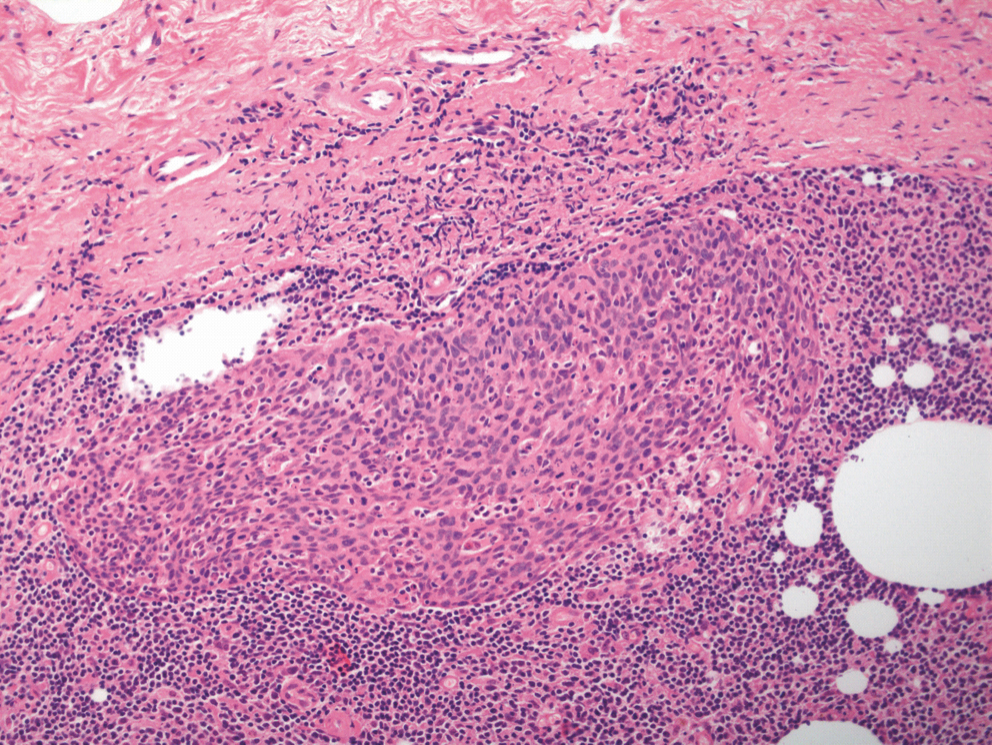
**Micrometastasis of LELC in right inguinal lymph node, H&E 100×**.

The total specimen measured 6.5:3:1 cm, the margins of resection were inconspicuous, and the oval tumorous lesion itself measured 1.5:1 cm. Histology revealed solid non-keratinizing sheets of tumor cells with high numbers of intraepithelial and stromal lymphocytes (Figure 1). The epithelial component did not show squamous or glandular differentiation, so the patient was diagnosed with lymphoepithelial- like carcinoma of the vulva (Figure 2). The depth of infiltration measured 2 mm, and there were no signs of angioinvasion or perineural spread. The lesion was completely resected. After processing the sentinel node in a complete series we found one micrometastasis of the same tumor type with a diameter of 1 mm (Figure 3). The staging was summarised pT1b, pN1mi(sn), L0, V0, R0, G3.

The antibodies used are listed in Table 1. EBV was analysed with in-situ hybridisation (ISH) using EBER probe, ready to use, Ventana. Staining for IHC and ISH was processed on Ventana Benchmark automated immunostainer (Ventana Medical Systems Inc. Tucson AZ 85755, USA).

**Table 1:** List of antibodies used for Immunohistochemistry.

ANTIBODY	CLONALITY	LOT	DILUTION	PRODUCER
Pancytokeratin	polyclonal rabbit		1:800	DAKO, Glostrup, Denmark

CK 5/6	monoclonal mouse	D5/16B4	1:20	DAKO, Glostrup, Denmark

p63	monoclonal mouse	4A4	ready to use	Ventana, Strasbourg, France

p16	monoclonal mouse	E6H4	ready to use	mtm labs, Heidelberg, Germany

CD3	polyclonal rabbit		1:100	DAKO, Glostrup, Denmark

CD4	monoclonal mouse	4B12	1:50	DAKO, Glostrup, Denmark

CD5	monoclonal mouse	4C7	1:100	Novocastra, Newcastle UK

CD8	monoclonal mouse	C8/144B	1:100	DAKO, Glostrup, Denmark

CD20	monoclonal mouse	L26	1:600	DAKO, Glostrup, Denmark

CD138	monoclonal mouse	MI15	1:50	DAKO, Glostrup, Denmark

TIA-1	monoclonal mouse	TIA-1	1:200	Biocare, CA, USA

CD56	monoclonal mouse	BC56C04	1:50	Biocare, CA, USA

Synaptophysin	monoclonal rabbit	SP11	ready to use	Ventana, Strasbourg, France

Chromogranin	monoclonal mouse	DAK-A3	1:500	DAKO, Glostrup, Denmark

MLH-1	monoclonal mouse	G168-15	1:50	BD Biosciences, MD, USA

MSH-2	monoclonal mouse	FE11	1:100	Calbiochem, NJ, USA

MSH-6	monoclonal mouse	44/MSH6	1:200	BD Biosciences, MD, USA

HPV	monoclonal mouse	BPV-1/1+CAMVIR	ready to use	Biocare, CA, USA

Immunohistochemistry showed positivity for Cytokeratin 5/6 and p63 indicating basal cell differentiation of the tumor. Wide spectrum cytokeratin did not react as strong and neuroendocrine markers like CD56, Synaptophysin und Chromogranin A were negative. Intraepithelial Lymphocytes were predominantly T-Lymphocytes, strongly staining for CD3, CD5 and CD8 with a positive CD8/CD4 ratio. The stromal lymphocytes mainly reacted positive for CD20, only a few with CD3 and CD8. MLH-1, MSH-2 and MSH-6 had clear nuclear expression. Staining for p16 was evenly strong but HPV was not detected by immunohistochemistry, the in-situ-hybridisation of EBV was negative. IGH and T/gamma rearrangement of the lymphocytic tumor component proved polyclonality.

### Discussion

Lymphoepithelioma-like carcinomas in extranasal or extrapharyngeal sites are uncommon tumors, and are rarely diagnosed in the gastrointestinal tract, the breast and the skin. Some scarce reports exist about LELC as a gynaecological neoplasm. LELC differs from its pharyngeal counterparts in some biological characteristics. Whereas the neoplasms of the upper aerodigestive tract are EBV-related, the most cases of LELC described so far were EBV negative [8-11], especially those in the skin and extracervical gynaecological sites [20-23]. Only very few reports could figure out a correlation between EBV and LELC of the uterine cervix [13,14]. With a negative EBV in-situ hybridisation this pathway could be excluded for our patient. In the present case histology apart from the main lesion showed intact squamous epithelium, there were no signs of vulvar intraepithelial neoplasia (VIN). Despite of the fact of a strong p16 positivity, immunohistochemistry of HPV was negative. There is some controversy about HPV related LELC [25,13,14], but a few reports give evidence for this possible pathogenesis in the uterine cervix [8,10]. Lichen sclerosus is a further precursor lesion of vulvar carcinoma, but we did not find lichenoid changes in the stromal compartments of the excision specimen. In this case of LELC, tumor associated lymphocytes were typically split up in T-lymphocytes dominating in the intraepithelial component, and B-lymphocytes and some plasma cells in the stromal areas.

Intestinal LELC frequently are microsatellite instable tumors, but normally these patients do not feature a clinical history of hereditary cancer. With strong positivity of MLH1, MSH2 and MSH6 this tumor can be assumed to show microsatellite stability.

Though the lesion was not advanced, one micrometastasis of 1 mm could be detected by sentinel node technique. Lymphoepithelioma-like carcinomas of the skin and the female genital tract are supposed to be associated with a better clinical outcome than their nasal or pharyngeal counterparts [1], but concerning local spread, angioinvasion and lymph node metastasis the results might not be so diverse [6,24]. Lymphoepithelial carcinomas of nasal or pharyngeal origin are sometimes detected by their local lymph node metastases exhibiting only small primaries, so early lymphatic angioinvasion is evident. Except for the present case there were only three reports about vulvar lymphoepithelioma-like carcinomas so far [5,7]. Two of them were locally excised without lymph node staging, and one patient had a long observation period and suffered from advanced disease with well known metastases before treatment [6].

## Conclusions

Summarised this could mean that apart from the pathogenesis, malignancy of vulvar LELC might not be so different from lymphoepithelial carcinoma of the nasal tract. Sentinel lymph node technique is appropriate for vulvar carcinomas and gives a good idea about aggressiveness and local spread. In this case of vulvar LELC sentinel technique indicates early metastasising potential, and leaves some reasonable doubts about the consistently maintained favourable outcome of this entity.

## Consent

Written informed consent was obtained from the patient for publication of this case report and any accompanying images. A copy of the written consent is available for review by the Editor-in-Chief of this journal.

## List of Abbreviations

EBV: Epstein-Barr virus; HPV: human papilloma virus; LELC: lymphoepithelioma like carcinoma; NOS: no other specification; VIN: vulvar intraepithelial neoplasia.

## Competing interests

The authors declare that they have no competing interests.

## Authors' contributions

HB conceived the case presentation. SH carried out histology and immunohistochemistry. CG participated in merging clinical data. FR carried out surgical therapy. WS conceived surgical and oncologic treatment. GW performed molecular analyses. All authors read and approved the final manuscript.
